# Translational landscape and protein biogenesis demands of the early secretory pathway in *Komagataella phaffii*

**DOI:** 10.1186/s12934-020-01489-9

**Published:** 2021-01-20

**Authors:** Troy R. Alva, Melanie Riera, Justin W. Chartron

**Affiliations:** 1 Department of Bioengineering, University of California, Riverside, 92521 United States of America; 2 Present Address: Protabit LLC, 1010 E Union St Suite 110, Pasadena, California 91106 United States of America

**Keywords:** Ribosome profiling, Protein secretion, Resource allocation, *Pichia pastoris*

## Abstract

**Background:**

Eukaryotes use distinct networks of biogenesis factors to synthesize, fold, monitor, traffic, and secrete proteins. During heterologous expression, saturation of any of these networks may bottleneck titer and yield. To understand the flux through various routes into the early secretory pathway, we quantified the global and membrane-associated translatomes of *Komagataella phaffii*.

**Results:**

By coupling Ribo-seq with long-read mRNA sequencing, we generated a new annotation of protein-encoding genes. By using Ribo-seq with subcellular fractionation, we quantified demands on co- and posttranslational translocation pathways. During exponential growth in rich media, protein components of the cell-wall represent the greatest number of nascent chains entering the ER. Transcripts encoding the transmembrane protein *PMA1* sequester more ribosomes at the ER membrane than any others. Comparison to *Saccharomyces cerevisiae* reveals conservation in the resources allocated by gene ontology, but variation in the diversity of gene products entering the secretory pathway.

**Conclusion:**

A subset of host proteins, particularly cell-wall components, impose the greatest biosynthetic demands in the early secretory pathway. These proteins are potential targets in strain engineering aimed at alleviating bottlenecks during heterologous protein production.

As microbial cell factories, yeasts offer many advantages for recombinant protein production including their natural properties and potential in synthetic biology. Yeasts grow rapidly to high densities in inexpensive media and are resistant to physical and chemical stress [1]. They also have an endomembrane system that is fundamentally conserved with higher eukaryotes [2]. This oxidative environment supports glycosylation and subsequent glycan modification, folding using ATP-driven molecular chaperones and protein disulfide isomerases, and protein quality control [3]. Compared to mammalian cells, yeasts have simpler genomes and can be more easily characterized and modified [4]. Combine these features with tools such as CRISPR/cas9, and the range of tractable species is expanding [5, 6]. *Komagataella phaffii* (one of two species previously known as *P. pastoris* [7–9]) stands out as a host for recombinant protein expression due to its high secretion capacity, its ability to metabolize methanol as its primary carbon source, its safety record as a source of biologics, and its extensive literature compared to other non-model yeasts [10, 11]. Thus, *K. phaffii* is an ideal chassis to rapidly implement changes designed to improve protein expression and secretion [4]. Indeed, recent work in *K. phaffii* has focused on systems-level analysis [12] and implementing design approaches of synthetic biology such as molecular parts lists and strain engineering [13, 14]. Such changes may accelerate product development and allow cheap, local production of pharmaceuticals [15, 16].

Identifying and relieving protein biogenesis bottlenecks is one strategy to improve yields of high-value, recombinant proteins [1, 17]. For secreted proteins expressed in *K. phaffii*, an early bottleneck is the translocation of newly made proteins from the cytoplasm into the lumen of the endoplasmic reticulum (ER) [18, 19]. Yeasts have multiple pathways for translocation, which use partially overlapping sets of biogenesis factors (reviewed in [2]). In the major pathway into the ER, translocation occurs through a membrane-embedded protein complex called the *sec* translocon. At least three major translocons exist in yeasts (the Ssh1 complex; two Sec61 complexes with, and without, Sec62p, Sec63p, Sec66p and Sec71p), which can accept proteins as they are synthesized by ribosomes (cotranslationally) or after synthesis of the polypeptide chain is complete (posttranslationally). Besides translocon architecture, co- and posttranslational pathways differ in their reliance on cytosolic molecular chaperones [20, 21]. Translocons bind hydrophobic amino acid motifs, called signal peptides, found at the amino termini of secreted proteins [22]. Some signal peptides are dependent upon a cytosolic factor, the Signal Recognition Particle (SRP), and the ER-bound SRP receptor to engage a translocon [23]; these tend to be longer or more hydrophobic than SRP independent signals [24, 25]. Binding of a signal peptide to a translocon opens the channel and allows the rest of the protein to pass into the lumen. In addition to secreted proteins, the *sec* translocon is a major point of entry for integral membrane proteins of the endomembrane system [26]. Integral membrane proteins that use a *sec* translocon require SRP for targeting to the ER over mitochondria [24].

For any production host, ribosomes, molecular chaperones, and *sec* translocons represent limited pools of resources that are distributed between heterologous proteins and the host proteome [27–29]. Unlike resources that are replenished enzymatically (like aminoacyl-tRNAs), ribosomes, translocons and chaperones only act on a single nascent chain at a time. While in use, they are sequestered and unavailable for other tasks. Although computational models that approximate these effects exist for bacteria [30], the complexity of eukaryotic translation is insufficiently understood to predict these allocations from transcriptomics alone. Accurate accounting of these resources could allow strains to be engineered in ways to relieve bottlenecks specific to a target. The secretome of *K. phaffii* has been characterized under several conditions [31], but the precise biosynthetic requirements of each protein remain unknown. Sequence features of secreted proteins, like glycosylation motifs, allow approximation of their direct biosynthetic costs such as ATP, carbohydrates, disulfide bonds, or GPI-anchors [32]. Per molecule costs can be coupled with measurements of gene expression to identify most expensive host proteins. Deletion of these proteins improves yields of secreted heterologous proteins in mammalian systems [33, 34]. However, while these analyses account for demands on global resources, they are limited by insufficient experimental data which links gene products to specific biogenesis subnetworks. For instance, overloading cotranslational translocons could limit secretory yields even if metabolic demands are met and posttranslational translocons are available. Quantification of global ribosome, cotranslational translocon and SRP use is available for *S. cerevisiae.* [24, 35, 36] However, these measurements are unavailable for other industrially significant species, including *K. phaffii.*

Which host proteins sequester the most biogenesis machinery in the early secretory pathway of *K. phaffii*? Which host genes produce the most nascent chains, competing for chaperones and sorting factors within the endomembrane system? To answer these questions, we quantified active translation globally and at the surface of the ER or mitochondria. Our analysis reveals the set of proteins that enter the secretory pathway cotranslationally and predicts the set that enter posttranslationally. In each set, we estimate demand for ribosomes and translocons. We distinguish between resources that act on a per nascent chain basis from machinery that is utilized based on elongation time.

## Materials and methods

### Strains and culture conditions

All experiments were performed using *Komagataella phaffii* GS115 (Invitrogen). For each Ribo-seq biological replicate, 500 ml liquid cultures of YPD (1% yeast extract, 2% peptone and 2% glucose) were grown to an $\hbox {OD}_{\mathrm{600\,nm}}$ of 2 at $30^{\circ }\hbox {C}$ with shaking in baffled 2 l flasks. Cells were harvested by vacuum filtration through a $0.8\,\upmu \hbox {m}$ filter. Immediately after filtering, cells were scraped off the filter using a chilled scoopula and submerged in a 50 ml conical tube containing liquid nitrogen. When indicated in order to match conditions of *S. cerevisiae* fractionated Ribo-seq data [35], cycloheximide (CHX) was added to $100\,\upmu \hbox {g}\,\hbox {ml}^{-1}$ for 3 min prior to harvesting. CHX treatments longer than a few minutes can alter ribosome abundance near the start of transcripts [37]. Short incubations with CHX enhance targeting of translocation competent ribosome-nascent chain complex while not perturbing non-secretory polysomes [36].

### Lysis and subcellular fractionation

Cells were lysed in either soluble lysis buffer ($50\,\hbox {mM}$ MOPS, $25\,\hbox {mM}$ potassium hydroxide, 100 mMolar potassium acetate, $2\,\hbox {mM}$ magnesium acetate, $1\,\hbox {mM}$ dithiothreitol and $100\,\upmu \hbox {g}\,\hbox {mL}^{-1}$ CHX) or membrane lysis buffer (soluble lysis buffer with 1% Triton X-100). Lysis buffers for each sample were frozen by adding $2\,\hbox {ml}$ dropwise to a $50\,\hbox {ml}$ conical tube containing liquid nitrogen. For each biological replicate, $\frac{2}{3}$ frozen cells were mixed with 2 ml frozen soluble lysis and the remaining $\frac{1}{3}$ were mixed with $2\,\hbox {ml}$ frozen membrane lysis buffer. Cell fractions were pulverized for $2\,\hbox {min}$ in a $50\,\hbox {ml}$ ball mill chamber with a single $2\,\hbox {cm}$ steel ball (Retsch) and collected into $1.5\,\hbox {ml}$ conical tubes. After thawing, lysates were centrifuged at 20,000×*g* for 10 min. Supernatants from samples lysed with membrane lysis buffer were collected and used as “total” fractions. Supernatants from samples lysed with soluble lysis buffer were collected and used as “soluble” fractions. The pellets from sample lysed with soluble lysis buffer were resuspended in $2\,\hbox {ml}$ membrane lysis buffer and centrifuged. The supernatants were collected and used as “membrane” fractions. Triton-X 100 was added to 1% in soluble fractions, so that all three fractions were in equivalent buffers.

### Ribo-Seq

Lysed samples were digested using 40 U of ribonuclease A (Ambion) for $1\,\hbox {h}$ at room temperature. Digested samples were layered on a 10 to 50% sucrose gradient prepared in $50\,\hbox {mM}$ Tris pH 7.5, $200\,\hbox {mM}$ sodium chloride, and $2\,\hbox {mM}$ magnesium acetate case using a Gradient Master (Biocomp). Gradients were centrifuged at $39,000\,\hbox {rpm}$ for $2.5\,\hbox {h}$ in a TH-641 rotor (Thermo). After centrifugation, gradients were fractionated using a Piston Gradient Fractionator (Biocomp) and monosome peaks were retained. Total RNA was extracted using a standard phenol-chloroform method and alcohol precipitated. Ribosome protected footprints, corresponding to (18 nt to 34 nt), were excised from a TBE urea gel. RNA was collected from excised gel fragments using RNA gel extraction buffer ($300\,\hbox {mM}$ sodium acetate, $1\,\hbox {mM}$ EDTA, and 0.25% SDS), precipitated, and resuspended in water containing $20\,\hbox {U}/\hbox {ml}$ SUPERase•In (Invitrogen).

Purified fragments were used to prepare sequencing libraries as described in [38] with some modification. Linker ligations were allowed to proceed for 4 h, and afterwards, samples were pooled and purified by TBE-urea PAGE. The pooled library was depleted of ribosomal RNA using the Ribo-Zero Gold rRNA Removal Kit (Illumina), following manufacturer’s instructions. Reverse transcriptions were performed using SuperScript II (Invitrogen). After circularization, PCR amplification and TBE PAGE purification, libraries were quantified using a Qubit 2.0 Fluorometer (Invitrogen) and sequenced using a HiSeq 4000 (Illumina.) Linker sequences were trimmed and libraries were demultiplexed using Cutadapt [39].

### Long read RNA sequencing

Cells were grown in YPD at 30 °C with agitation to an $\hbox {OD}_{600\,\mathrm{nm}}$ of 2 and harvested by centrifugation. Total RNA was obtained using a Direct-Zol kit (Zymo Research). Cells were vortexed with glass beads for 2 min during incubation with TRI reagent. After purifying RNA, a library was prepared using a PCR-cDNA kit according to manufacturer’s instructions (SQK-PCS109, Oxford Nanopore Technologies) and sequenced using a minION R9.4.1 flow cell. Base calling was performed using Guppy (Oxford Nanopore Technologies).

### Transcript assembly

A novel transcriptome was assembled using data derived from Ribo-Seq, long-read RNA-Seq, and a prior genome sequence of strain GS115 [40]. A flowchart of the annotation pipeline is provided in Figure S2c. Ribo-seq reads and long reads were aligned to the reference genome using HISAT2 [41] and Minimap2 [42] respectively. Stringtie version 1.3.6 was used to assemble transcripts from Ribo-seq data, with reads mapping to each strand processed separately [43]. Pinfish was used to assemble transcripts from long reads (Oxford Nanopore Technologies). After transcript assembly, PASA [44] was used to combine the Stringtie and Pinfish models into a single transcriptome. Transdecoder [45] was then run twice: first, to identify candidate coding regions with PASA model with a lower limit of 100 amino acids, and second, to identify coding regions in just the Stringtie model with a lower limit of 40 amino acids. The latter run has a reduced risk of misannotating start codons in the 5′-UTR. Transdecoder annotated transcripts from $\hbox {Transdecoder}_{\mathrm{PASA}}$ were used to train GlimmerHMM [46] and CodingQuarry [47], which were used to provide de novo predictions in the genome. EVidenceModeler [48] was used to incorporate predictions from PASA, $\hbox {Transdecoder}_{\mathrm{Stringtie}}$, $\hbox {Transdecoder}_{\mathrm{PASA}}$, GlimmerHMM and CodingQuarry. File processing, UTRs, and tRNAs annotations were provide by the update utility in the Funannotate package [49].

### Mapping of ribosome protected reads to codons and masking

Ribo-seq reads were mapped to the genome of *Komagataella pastoris* GS115 [40] using HISAT2 [41, 50]. Alignments were converted from SAM to sorted and indexed BAM files using Samtools and only included reads with mapping quality threshold of 60 [51]. Mapped reads were loaded into R using the GenomicAlignments package from Bioconductor[52] and converted to their 3′ end positions before determining p-site offsets. P-site offsets were determined using the RiboProfiling package in Bioconductor [53]. Each read was mapped to a single codon. Masking files were created by first parsing the coding sequence (CDS) annotation file associated with the reference genome into a fasta file simulating every possible 28 nt combination (approximate length of a ribosome protected mRNA fragment). This fasta file was then aligned to reference genome twice, once to only include reads with mapping quality greater than or equal to 60 (unambiguously assigned), and another to include all reads (ambiguously assigned). Both alignment files were used to generate reads per codon per gene (RPCPG) data tables. The unambiguously assigned reads were subtracted from ambiguously assigned reads and codons with a nonzero difference were included in mask. The first and last five codons in genes’ open reading frames (ORFs) were masked to correct for variable read quality at the beginning and ending of transcripts inherent to Ribo-Seq [54].

### Metagene correction and quantification of metabolic demand

Read counts were normalized at the codon level using a metagene analysis that provides a global profile for each data set. First, for each ORF, reads at each codon position were scaled by the average reads per codon mapped ORF. Then, for codon position, either a mean or median value was calculated from all ORFs using the following scheme: for positions 1 to 100, a rolling mean with a window of 10 codons; for positions 100 to 1000, a rolling mean with a window of 100; for positions 1000 and onward, a rolling median with a window of 1000. In calculating corrected transcripts per million (cTPM), codon read counts were scaled by dividing the metagene-derived value at that position and normalized by their pseudo gene lengths (theoretical gene length minus number of masked codons) and a per million scaling factor unique to each data set. In calculating ribosomes per million (cRPM), a ribosome scaling factor was created for each gene by dividing the sum of the metagene-derived values at all codon positions by the sum of smoothed reads per codon with the mask applied (a gene with zero masked codons will have a ribosome scaling factor equal to one, while a gene that contains masked codons will have a scaling factor greater than one). The ribosome scaling factor is multiplied by unmasked gene read counts and normalized by a per million scaling factor unique to each data set to give RPM. Membrane enrichment is quantified for each gene as the $\text{log}_{2}$ ratio of membrane cTPM scores or total cTPM scores to soluble cTPM scores.

### Classification and annotation of ORFs

Gene names were hierarchically assigned to novel *K. phaffii* transcripts through homology. Firstly, transcripts were assigned names inherited from *S. cerevisiae* using BlastP [55] with an expected value less than 1e−5. For genes that were not predicted to be homologous, gene names were assigned common names using EggNOG 4.5 [56] using a taxonomic scope limited to ascomycetes. Genes that did not share homology with *S. cerevisiae* or known ascomycetes were assigned names inherited from *K. phaffii* GS115 [40] using BlastP with expected values less than 1e−5. Novel genes that were not assigned names using methods above were named after the moniker given during transcript assembly.

ORFs were classified by function, cellular location, and sequence features using various prediction software. Functions were assigned ontologically using clusters of orthologous groups (COG) and were prepared using EggNOG 4.5 [56]. Vironoi tessellations were created to quantitatively map the biosynthetic composition of these functions using COGs and expression metrics derived from Ribo-Seq cTPM [57]. DeepLoc was used to predict the subcellular localization associated with ORF products [58]. Sequence features such as signal sequences, transmembrane domains (TMD), and GPI anchors were identified using SignalP 5.0 [59], TOPCONS [60], and predGPI [61] respectively.

### *S. cerevisiae* analysis

Ribo-seq data for total protein synthesis were taken from [62], and data obtained from soluble or membrane-bound ribosome fractions were obtained from [35]. All data were processed in the same way as *K. phaffi* using the S288C reference genome R64-2-1 [63].

## Results

### Ribo-seq and long-read RNA-seq improve open reading frames annotations

**Fig. 1:**
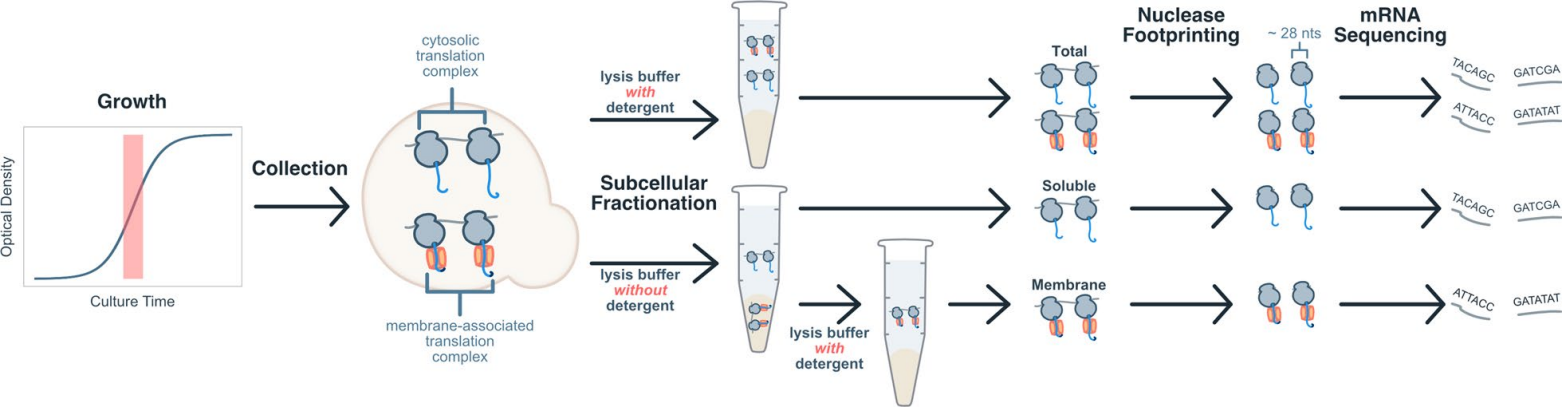
Overview of Ribo-seq and subcellular fractionation. Ribosomes (grey) bound to a translocon (red) are only solubilized in the presence of detergent. The total sample has footprints originating from both membrane-bound and free-floating ribosomes. The soluble fraction is enriched in footprints from free-floating ribosomes. The membrane fraction is enriched in footprints from membrane-bound ribosomes

We sought to globally quantify several aspects of protein synthesis in *K. phaffii* GS115. We asked which genes were responsible for sequestering limited biosynthetic resources, such as ribosomes and ER translocons. We also asked which genes were responsible for producing the most nascent chains, which is critical for predicting amino acid usage, as well as modifications that act on a per chain basis (i.e., N-terminal acetylation, GPI anchoring, vesicular sorting). Ribo-seq provides a snapshot of protein translation, allowing us to answer both of these questions [64]. It is a high throughput sequencing technique used to infer ribosome abundance at each codon of each transcript. In Ribo-seq, a non-specific ribonuclease generates 20 to 22 nt or 28 nt to 30 nt “footprints” of ribosome-protected mRNA depending on the translational conformation of the ribosome [65], which are then sequenced. We performed a series of Ribo-seq experiments to capture global translation and translation on the surface of organelles (Fig. 1). Our data sets captured footprint lengths from 15 to 42 nt (Additional file 1: Figure S1a). Nearly all (99%) footprints mapped within open reading frames (ORFs). Our profiling data also indicate active translation through the appearance of three nucleotide periodicity in read depth that is preserved across the transcriptome (Additional file 1: Figure S1b).

**Fig. 2:**
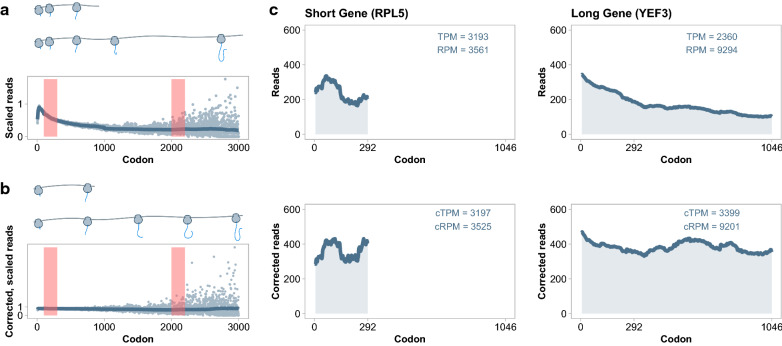
Corrections applied to Ribo-seq data. **a** Ribosome-protected read counts at each codon were scaled by the total reads mapping to the ORF. Dots represent individual codons, and the line represents a composite of rolling means and medians see Methods. Regions in orange are the same width and are used to demonstrate that masked codons at the beginning of ORFs have a greater influence of calculated expression than masked codons at the end of ORFs. **b** Data from **a** after metagene correction. **c** Comparison of ribosome-protected reads per codon for highly expressed genes of different length. TPM for *RPL5* gene is approximately 135% greater than TPM for *YEF3* while producing approximately 38% as many ribosome-protected reads. After metagene correction cTPM scores are similar preserving the same difference in ribosome sequestration

**Fig. 3:**
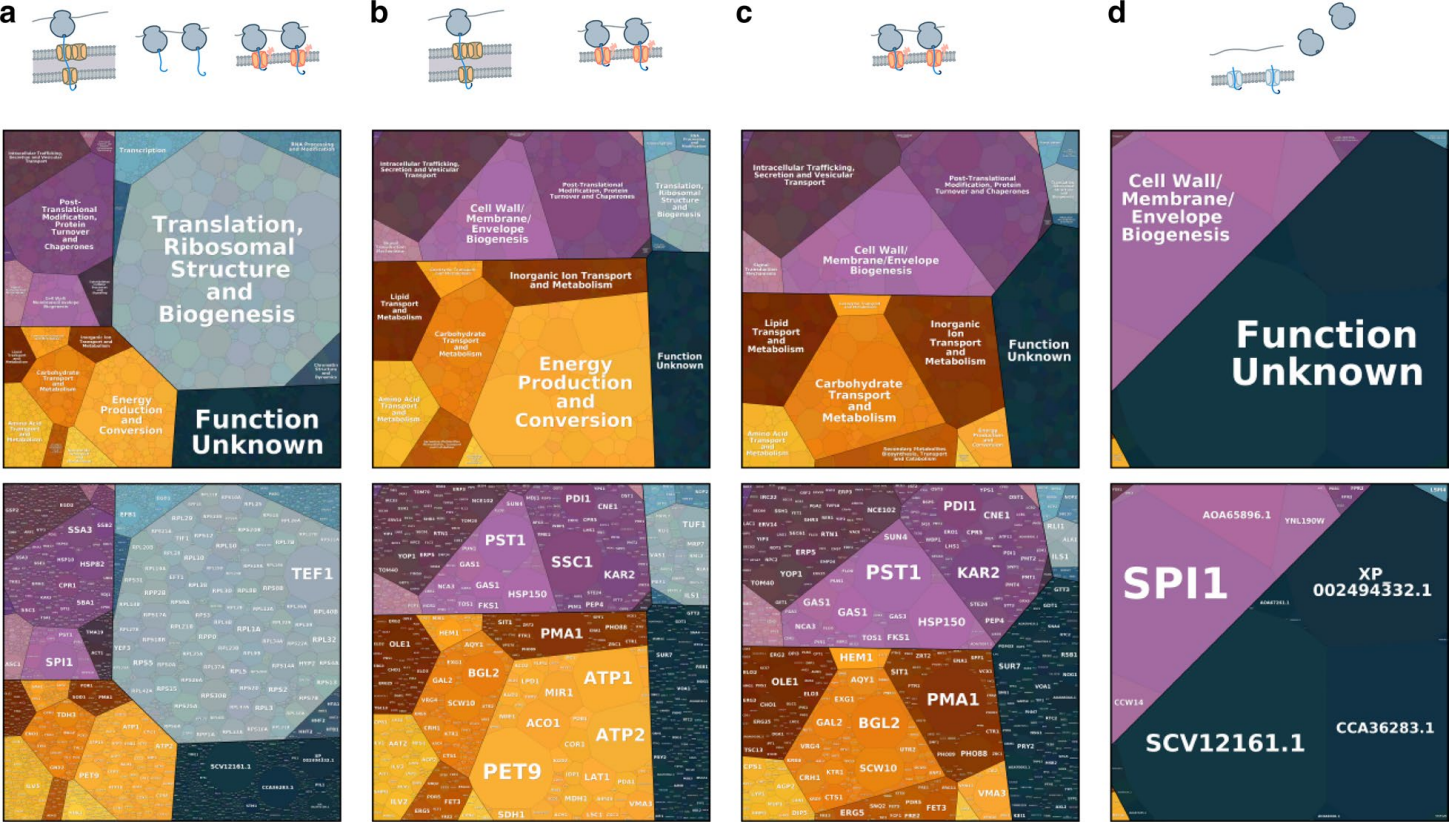
Protein expression and trafficking in *K. phaffi*. Tessellations are calculated using cTPM from the total fraction of a CHX treated culture and represent relative quantities of nascent chains produced from each gene. **a** Nascent chains produced by all ribosomes. **b** Nascent chains from genes showing twofold membrane enrichment. This includes mitochondrial and ER destined proteins. **c** Nascent chains from genes showing twofold membrane enrichment that are not predicted to be mitochondrial. **d** Nascent chains from genes showing less than twofold membrane enrichment but with a predicted ER signal sequence

**Fig. 4:**
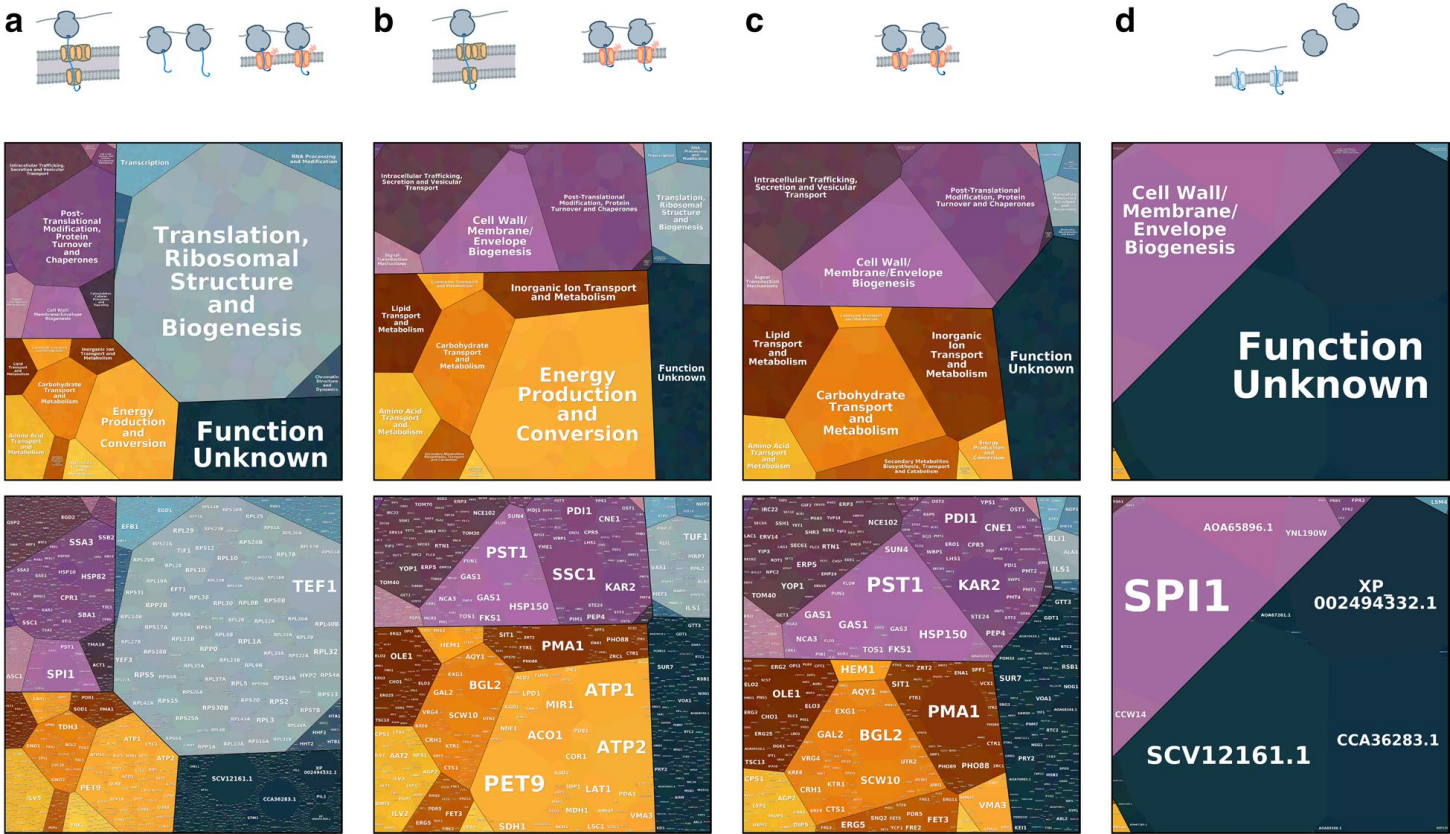
Comparison of translation from samples of membrane-bound and soluble fraction. Values are calculated using fractions obtained after incubation with CHX

**Fig. 5:**
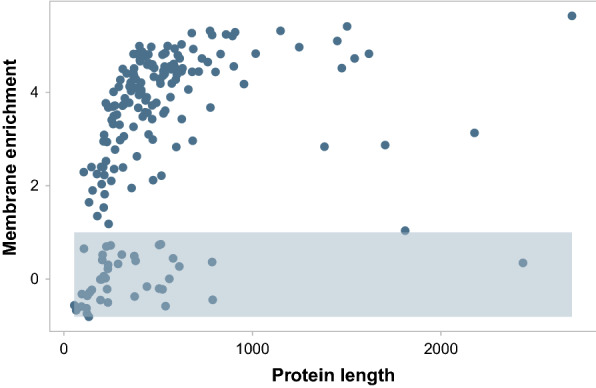
Nascent peptide length and membrane enrichment for secreted, lumenal, or GPI-anchored proteins. Proteins have a predicted N-terminal signal sequence. GPI anchors are included. The shaded box is drawn over genes with less than twofold membrane enrichment, which are considered posttranslationally targeted

We noticed that ribosome-protected read patterns were often inconsistent with prior annotations of open reading frames (Additional file 2: Figure S2a). At many loci, Ribo-seq appeared to indicate that translation began at an alternate start codon. Inaccuracies in ORF structure are problematic, since the length of a reading frame is a critical parameter used for quantifying translation and the position of the start site is used in correction using global profiles (see below). We therefore sought to improve the GS115 annotation using Ribo-seq. Several methods that rely solely on Ribo-seq to annotate structure rely on the three nucleotide periodicity of reads to define reading frames [66]. They require substantial coverage for each gene; however, sparse Ribo-seq coverage could still support re-annotation if it were treated like stranded RNA-seq data. Moreover, de novo open reading frame predictors can be trained using verified translational start sites, and so improving the accuracy of annotations for a subset of the transcriptome was expected to improve overall prediction accuracy. We therefore adapted consensus methods used in gene prediction and annotation with standard RNA-seq data, with optimizations for fungi [48, 49]. Our approach uses Ribo-seq to construct transcript models, which are then used to train several de novo annotators.

Like other yeasts, *K. phaffii* has short intergenic sequences, leading to overlapping untranslated regions (UTRs), even on transcripts encoded on the same DNA strand. As a result, methods that construct transcripts from short-read sequencing merge data from adjacent genes into a single transcript. We therefore collected long-read data using Oxford Nanopore PCR-cDNA sequencing and developed a pipeline to integrate Ribo-seq, long-read RNA-seq, and de novo gene prediction (Additional file 2: Figure S2b, c). Our annotation is provided as Additional file 3. ORFs that were fully covered by Ribo-seq data were allowed to be as short as 40 amino acids, increasing the number of annotated genes compared to other annotations of *K. phaffii* (Table 1) [40, 67, 68]. Homologs between our annotation and prior annotations are provided as Additional file 4. Our annotation adjusted the translational start site of about 10% of ORFs compared to each previous model. Overall, Ribo-seq reads were mapped to 5303 genes in *K. phaffii* in the assembly presented here. We have named genes based on homology to prior annotations, to *S. cerevisiae* and to other ascomycetes.

**Table 1:** Comparison of ORF annotations

Annotation^a^	Total ORFs	Homologs^b^	Length differences^c^
Current study	5329		
GS115 (PRJNA304976)	5064	5035	514
GS115 (PRJEA37871)	5040	5100	697
CBS7435 (PRJEA62483)	5291	5198	604

^a^NCBI bioproject numbers located in parenthesis

^b^BlastP matches from current study to prior study

^c^Number of homologs with different predicted lengths

### Translational landscape of *K. phaffii*

Each read in Ribo-seq originates from a translating ribosome. Thus, by comparing the distribution of reads, we can answer our first question and identify which transcripts sequester ribosomes and ribosome-associated factors, like the *sec* translocon. As a method to predict the abundance of polypeptide chains, Ribo-seq has greater sensitivity than mass spectrometry, and more closely matches measurements of protein abundance than RNA-seq [69]. To answer our second question, the number of nascent polypeptide chains produced per unit time can be approximated using a modified form of the transcripts per million (TPM) metric used in RNA-seq. TPM has advantages over other metrics (RPKM or FPKM) for its intuitive interpretation during differential analysis and for its congruence with proteomics [70, 71]. In RNA-seq, reads are generally long enough to be unambiguously mapped to the transcriptome, and they can be assumed to equally cover a transcript. In Ribo-seq, however, these assumptions do not hold, and biases due to ambiguous mapping and unequal coverage must be corrected.

**Fig. 6:**
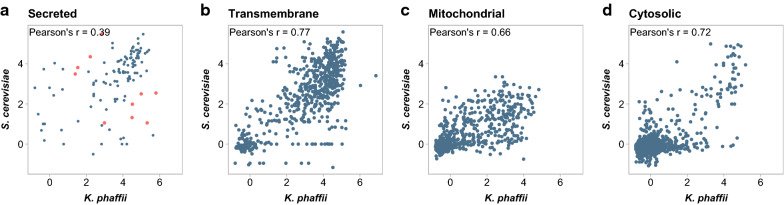
Correlation of membrane enrichment scores between species. Scores are deteremined using the membrane-bound and soluble fractions of ribosomes from cultures treated with CHX. **a** Enrichment scores restricted to signal sequence bearing proteins. Contrast dots represent genes found in Table 2. **b** Enrichment scores restricted to non-mitochondrial transmembrane proteins. **c** Enrichment scores restricted to mitochondrial proteins. **d** Enrichment scores restricted to cytosolic proteins

**Fig. 7:**
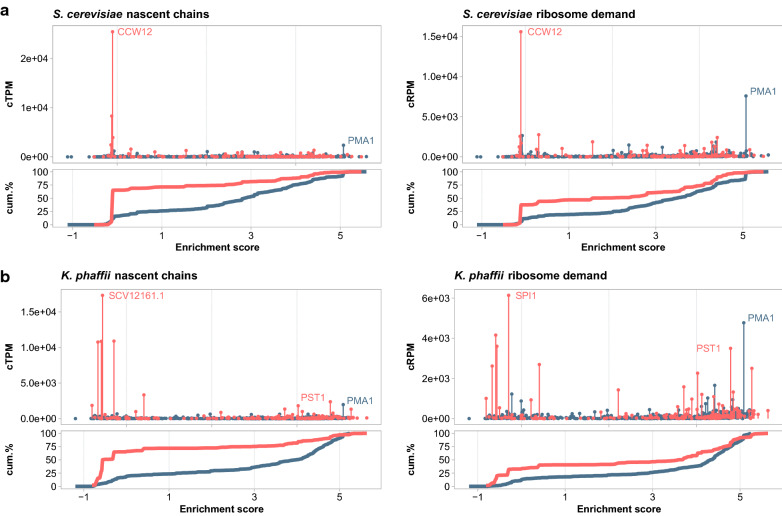
Demands imposed on secretion pathway. Blue lines represent membrane proteins and orange lines represent secreted, lumenal or GPI-anchored proteins. **a** Demands in *S. cerevisiae*. **b** Demands in *K. phaffii*

**Fig. 8:**
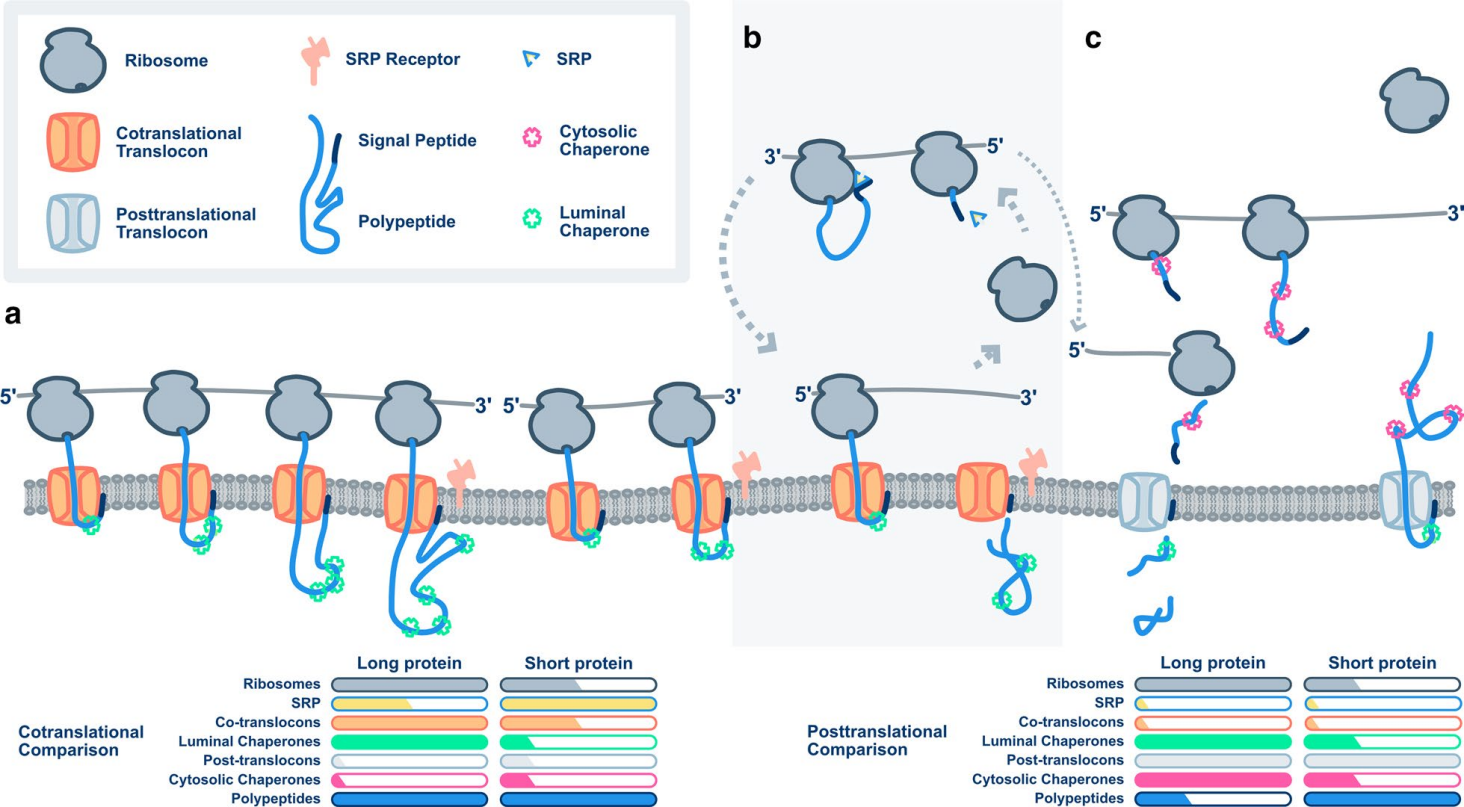
Demands imposed by different translocation pathways. **a** Cotranslational translocation of long protein and short proteins. **b** Translocation of short proteins which require both co- and posttranslational translocons **c** Posttranslational translocation

Ribosome protected fragments are small, 22 nt to 30 nt, and may map to multiple mRNA sequences when the transcriptome contains homologous stretches. Ambiguously mapped reads can be handled in one of several ways, often with shortcomings. Discarding multi-mapped reads [72–75] depreciates read counts for highly expressed genes. Randomly assigning reads to ORFs with equivalent percentage of alignment [64, 76, 77] overestimates read counts for lowly expressed genes. Here, we adapt the method of Taggart et al. [62], who used computational masks to exclude homologous segments of the predicted transcriptome. We calculated a mask over the *K. phaffii* transcriptome accounting for all possible 28 nt reads, excluding 3% of codon positions available. To estimate gene expression via TPM, reads must be scaled by ORF length. Unlike discarding or randomly assigning reads, masking adjusts the gene length to reflect mRNA positions available for analysis. However, masking alone is insufficient because ribosome protected reads are not evenly distributed across transcripts.

Ribosome-protected reads are more abundant near the 5′ end of ORFs [64, 78]. This effect may be due slower elongation rates at the beginning of translation [79] or abortive translation [62]. Regardless of the mechanism, the positional bias is observed in nearly every transcript and results in a global read profile that is conserved across the translatome (Fig. 2a). As a result, estimates of the expression of short ORFs will appear inflated (and long ORFs deflated), since only the ribosome-rich region of the global profile is sampled. We again adapt the method of Taggart el al. [62], where the positional bias is removed by scaling reads at each codon by the empirical global profile. (Fig. 2b). We use corrected TPM (cTPM), with masking and scaling, as a measure of the rate at which nascent chains are produced. For example, transcripts of *RPL5* and *YEF3* display similar numbers of ribosomes at the start of their ORFs (Fig. 2c), suggesting similar initiation rates. However, because *YEF3* is a longer ORF, its standard TPM is smaller than the TPM of *RPL5*. Here, we assume that if *RPL5* were as long as *YEF3*, then its translational profile will be similar to the global profile, resulting in similar cTPM scores.

While cTPM estimates the number of nascent polypeptide chains, it does not answer our question regarding ribosome sequestration. Longer transcripts sequester a greater number of ribosomes in order to produce the same number of nascent chains as a shorter transcript. If ribosomes accumulate near the start codon in vivo, then it is important to include this effect while measuring allocation. cTPM, therefore, is an inappropriate metric. If ribosome-protected reads could be unambiguously mapped to the transcriptome, then simple read counts estimate ribosome usage per gene. However, when masking is applied, the position of the mask becomes important (Fig. 2a, b). Two masks of the same length, applied at different positions, will hide different amounts of ribosomes based on the global profile. To correct for this, we introduce a ribosome scaling factor that accounts for masking of each gene. The factor represents the fraction of ribosomes expected to be observed when the gene-specific mask is applied to the global translational profile. We generate a new metric for each gene, corrected ribosomes per million (cRPM), which is practically equivalent to reads per million (RPM) in standard RNA-seq. In our example in Fig. 2c, cRPM and RPM are almost identical, as expected since there are no masks applied to *RPL5* or *YEF3*. Read counts, cTPM and cRPM for each gene in each dataset are provided as Additional file 5.

After applying corrections, we find that the majority of nascent chains synthesized in *K. phaffii* are from genes involved in translation, ribosomal structure and biogenesis (see Table 2 and Fig. 3a), as expected for log-phase growth. The majority of nascent chains encoded by genes of unknown function are predicted to be extracellular, where they are likely components of the cell wall. We consider endomembrane lumenal and secreted proteins to be those with (i) predicted N-terminal signal sequences, (ii) are not predicted to be localized to the mitochondria, and (iii) contain less than or equal to one transmembrane domain, as these are frequently GPI anchors. Some single-pass, type I transmembrane proteins will be misannotated by this definition. The number of genes containing these predictive features and the relative percentage of nascent chains they produce are summarized in Table 2. A majority of nascent chains for genes containing a signal sequence also contain GPI anchors, suggesting that this structural class represents the majority of products that will be processed by the secretory pathway.

**Table 2:** Nascent chains produced in *K. phaffii*

	Nascent chains (%)^a^	Genes (n)
Ontological functions		
Translation, rbosomal structure and biogenesis	44.0	366
Function unknown	11.0	1602
Post-translational modification, protein turnover and chaperones	9.0	409
Energy production and conversion	8.0	207
Intracellular trafficking, secretion and vesicular transport	4.0	382
Carbohydrate transport and metabolism	3.0	218
Cell wall/membrane/envelope biogenesis	3.0	85
Amino acid transport and metabolism	3.0	191
Transcription	2.0	355
RNA processing and modification	2.0	242
Predicted features of ER destined proteins		
Lumenal and secreted proteins^b^	8	266
GPI Anchors	79^c^	117
Transmembrane proteins^d^	7	960

^a^Nascent chains are percentage of the total cTPM represented by each category

^b^Total number of genes with an N-terminal signal sequence and may include a GPI anchor

^c^Percentage of nascent chains containing signal sequences that also contain a predicted GPI anchor

^d^Transmembrane proteins either have no signal sequence but one transmembrane domain (TMD), or two or more TMDs

### Biogenesis demands in the early secretory pathway

We next investigated the global demands for machinery needed for translocation into the ER. Subcellular fractionation was used to separate membrane-bound ribosomes from free floating, soluble ribosomes. Membrane-bound ribosomes were detergent solubilized, and then samples from both soluble and membrane fractions were subject to Ribo-Seq (Fig. 1). As in *S. cerevisiae*, libraries derived from the membrane fractions are enriched in ribosome-protected footprints originating from transcripts that encode proteins destined for the ER or mitochondria [35] (Fig. 4). Membrane enrichment scores were calculated as the $log_2$ ratio of cTPM for membrane and soluble fractions and were reproducible (Additional file 6: Figure S3a and provided in Additional file 5). The magnitude of membrane enrichment scores depends on the efficiency of fractionation, and if a gene falls below the diagonal line in Fig. 4, it will have a negative enrichment score. As in *S. cerevisae*, membrane enrichment scores are limited by the length of the ORF when transcripts encode signal-sequence bearing proteins [35, 36] (Fig. 5). This effect is due to a kinetic competition between trafficking rate and translation elongation rate. Fig. 5 also reveals that a membrane enrichment score of 2 effectively separates two populations, and so we define genes with scores greater than 2 as cotranslationally translocated into either the ER or mitochondria. The set of cotranslationally translocated nascent polypeptides is enriched for those involved in energy production and conversion, cell wall and membrane biogenesis, and various transporters (Fig. 3b). To assess entry into the ER, we filtered out transcripts encoding proteins predicted to localize in the mitochondria by DeepLoc (Fig. 3c). Finally, we define proteins that enter the ER through a posttranslational *sec* translocon as those having a predicted N-terminal signal sequence and less than twofold membrane enrichment (Fig. 3d). Posttranslationally trafficked membrane proteins rely on other mechanisms, such as the GET pathway [22].

A more diverse group of proteins enter the ER through cotranslational translocons than those that enter posttranslationally (Fig. 3c,d and Table 3). While the diversity of functions for proteins that enter the ER posttranslationally is relatively small (mostly unknown function and then cell wall and membrane biogenesis), we find that posttranslational translocation handles a majority of total nascent chains entering the ER. These genes encode primarily small proteins such as SCV12161.1p or cell wall proteins processed with GPI-anchors, such as Spi1p. Although its function is unknown, Spi1p is also predicted to be GPI-anchored, and both *SPI1* and *SCV12161.1* produce among most nascent proteins within the cell under conditions tested here (Fig. 3a). We then classified the genes of unknown function that entered the ER by their predicted final location. The majority of these gene products, approximately four fifths, are predicted to be localized extracellularly and have an unusual discrepancy between their relative ribosomal usage, nascent chains produced, and average gene length compared to unknown genes predicted to localize elsewhere (Additional file 7: Table S1).

**Table 3:** Comparison of translocon demands by ontological function

	Genes (n)	Nascent^a^ (%)	Ribosomes^b^ (%)
Cotranslationally translocated^c^			
Function unknown	261	8.00	11.0
Cell wall/membrane/envelope Biogenesis	41	7.00	12.0
Post-translational modification, protein turnover and chaperones	89	7.00	12.0
Carbohydrate transport and metabolism	114	7.00	9.0
Intracellular trafficking, secretion and vesicular transport	95	6.00	7.0
Inorganic Ion transport and metabolism	82	5.00	10.0
Lipid transport and metabolism	72	4.00	5.0
Posttranslationally translocated^d^			
Function unknown	30	36	11.0
Cell wall/membrane/envelope Biogenesis	10	15	10.0
Post-translational modification, protein turnover and chaperones	5	0	0.0

^a^Calculated as percent of total cTPM for all proteins predicted to be ER destined

^b^Calculated as percent of total cRPM for all proteins predicted to be ER destined

^c^Proteins with greater than twofold membrane enrichment and not predicted to be mitochondrial

^d^Proteins with less than twofold membrane enrichment and not predicted to be mitochondrial and contained a predicted signal sequence

### Comparing the translational landscape between *K. phaffii *and *S. cerevisiae*

Of the 5329 *K. phaffii* genes annotated here, 73% have a homolog in *S. cerevisiae*. Unlike *K. phaffii*, *S. cerevisiae* is thought to have undergone a whole-genome duplication, and so many *S. cerevisiae* genes have paralogs [80]. The influence of paralogy is evident in how these two species allocate translational throughput. We calculated of cTPM and cRPM in *S. cerevisiae* (Additional file 8) using prior data acquired under similar growth conditions [35, 62]. The overall distribution of cTPM by ontological category is similar between species (Additional file 9: Figure S4). Under the conditions tested here (glucose-containing rich media), *TEF1*, encoding translational elongation factor 1 alpha, is the most translated protein in *K. phaffii*. The TEF1 promoter is used to drive constitutive expression in *K. phaffi* [81], and our results suggest that the native TEF1 ORF is translated more than the ORFs linked to other promoters used for expression in glucose, such *GAP* (here, *TDH3*) and *PGK1* [11]. *S. cerevisiae* generates a similar amount of nascent chains to the same function, but it does so using a combination of its paralogous genes *TEF1* and *TEF2*. Unsurprisingly, Crabtree-positive *S. cerevisiae* generates three times more polypeptides involved in glycolysis and fermentation than *K. phaffii* (e.g., *ENO1/2, GPM1, FBA1, TDH2/3, TPI1, PGK1, PDC1, ADH1*).

Indeed, these two species also show divergence in energy production with regards to cotranslational mitochondrial import (Fig. 6). Our subcellular fractionation assay recovers all membrane-bound ribosomes, including those attached to the mitochondria. A greater number of nuclear-encoded mitochondrial proteins undergo membrane-localized translation in *K. phaffii*. Recovery of membrane associated mRNA strongly depends on active translation [35]. Therefore, less active translation of mitochondrially destined proteins may become reflected in lower membrane-enrichment scores.

We next asked whether ER translocation pathways are conserved between the two species. Between homologs, membrane enrichment scores correlated with a Pearson’s r of 0.85 (Additional file 6: Figure S3b). Genes encoding transmembrane proteins or cytosolic proteins which lack ER or mitochondrial targeting sequences had the highest correlation. Signal-sequence bearing proteins, including GPI-anchored proteins, however, had lower correlation (Fig. 6a). There were several genes which only showed cotranslational membrane enrichment in one species, and in some cases this was due to loss of a signal peptide in one of the homologs. The ten genes that showed the greatest difference in magnitude, while still showing evidence for membrane enrichment in both species, are reported in Table 4. Notably, this list includes *PDI1*, encoding an ER lumenal protein-disulfide isomerase that is essential for ER homeostasis. Mitochondrially localized proteins have greater membrane enrichment in *K. phaffii*, which may be related to the greater use of aerobic respiration compared to *S. cerevisiae* (Fig. 6*c*).

**Table 4:** Membrane enrichment for secreted, lumenal and GPI-anchored proteins in *K. phaffii* and *S. cerevisiae*

Gene	Product	*K. phaffii*	*S. cerevisiae*
Increased enrichment			
FLO9	Lectin-like protein, flocculin (isoform 2)	5.32	1.06
ZPS1	Putative GPI-anchored protein	5.80	2.54
SGA1	Sporulation-specific glucoamylase	4.49	1.32
BIG1	Cell wall beta-1,6-glucan level regulator	4.51	1.99
GDA1	Guanosine-diphosphatase	4.99	2.50
FLO9	Lectin-like protein, flocculin (isoform 1)	2.99	1.06
Decreased enrichment			
YKL077W	Uncharacterized protein	1.39	3.49
PDI1	Protein disulfide isomerase	2.21	4.35
MNL1	Uncharacterized protein	1.53	3.81
KRE5	Beta-1,6-glucan biosynthesis protein (isoform 2)	2.84	5.47

Finally, we explored the relationship between the burden imposed by production of polypeptide chains (cTPM), ribosome demand (cRPM) and translocation pathway (membrane enrichment score) for ER destined proteins within the two species (Fig. 7). In *S. cerevisiae*, most of these chains originate from a single gene, *CCW12*, while in *K. phaffii*, there are a wider variety of genes, with *SCV12161.1* being the most dominant. Strikingly, posttranslational targeting is used for about two-thirds of lumenal, secreted or GPI-anchored nascent chains in both species. *K. phaffii*, however, is distinguished by at least one major cell wall protein, Pst1p, which enters the ER cotranslationally. In both species, Pma1p is the dominant membrane protein passing into the ER. In terms of ribosome sequestration, the trend reverses; cotranslational translocation is responsible for sequestering two thirds of ribosomes used to produce secreted or GPI-anchored proteins. While *PST1* yields slightly more nascent chains than *PMA1*, *PMA1* is more than twice as long as *PST1* and sequesters 1.36 times more ribosomes. Thus, *PMA1* represents a significant burden to the secretory systems of both *S. cerevisiae* and *K. phaffii* as it is predicted to sequester more ribosomes, cotranslational translocons, and lumenal chaperones to synthesize and transport nascent chains into the ER.

## Discussion

The yields of engineered, recombinant proteins are restricted by bottlenecks in biogenesis [1]. Certain bottlenecks are metabolic, including insufficient ATP or other high-energy compounds, nucleotides for mRNA synthesis, amino acids, carbohydrates for glycosylation, and reducing equivalents. A promising systems-level approach to remove bottlenecks is to identify and delete host proteins with the greatest demand for metabolic resources. Indeed, the Lewis lab has elegantly demonstrated in CHO cells that deleting expensive proteins (in terms of ATP equivalents) increases the yield of heterologous secreted proteins [33, 34, 82]. Similar modeling of metabolic demand has been performed by the Nielsen lab for the secretome of *S. cerevisiae* [32]. Other bottlenecks are due to insufficient cellular protein biosynthetic machinery, such as polymerases, ribosomes, translocons, and molecular chaperones. Focusing on metabolic demand will likely relieve pressure on machinery with tightly coupled–and therefore accurately predicted–energetic requirements (e.g., cycles of translation elongation by the ribosome). However, it only approximates demand for chaperones and translocons, which gate entry into the ER. Compared to tightly coupled complexes, chaperones and translocons are ambiguous in their energetic demand. Chaperones perform cycles of binding and rebinding that depend on the folding pathways of client proteins [83]. Translocation into the ER is driven by ATP-hydrolyzing chaperones, translation elongation, or a combination of the two in a client dependent manner [84, 85]. Engineering of the early secretory pathway, such as the optimization of signal sequences for protein targeting [86] and reducing the effect of the ERAD system [19], provides varying degrees of success. These approaches are contingent on the complexity of the protein product and must be empirically optimized [87, 88]. Our data and analysis may augment these efforts by accounting for capacity of translation, co- and posttranslational translocation.

Despite the ability of Ribo-seq to accurately quantify gene expression, our study has several caveats that limit interpretation. First, we have only considered yeast undergoing log phase growth in liter scale, aerated shaking cultures using rich media. This design enabled comparison to several published data sets using *S. cerevisiae* that were collected under identical conditions [35, 62]. We chose strain GS115, a commonly used commercially available strain that is a histidine auxotroph (*his4*). Even under rich media with abundant extracellular histidine, this auxotrophy may influence gene expression compared to strains which supply *HIS4*. Future work involves quantifying demands at industrial scale in stirred bioreactors under induction of a heterologous protein. Second, we assume that elongation rates are relatively constant across genes. However, if the elongation rate is altered for a transcript, it may result in greater or fewer ribosome protected reads. We argue that on the whole, our assumption is valid, given that Ribo-seq accurately predicts mature protein stoichiometry [62, 89]. Third, Ribo-seq does not account for protein degradation; indeed, some proteins are cotranslationally ubiquitinated [90]. Our results should therefore not be interpreted as revealing steady-state protein levels in *K. phaffii*. However, our goal was to quantify the costs of protein synthesis, and so we argue that Ribo-seq is a more appropriate tool than mass spectrometry. Despite these limitations, our approach allowed us to interrogate protein translocation into the ER.

Most secreted proteins, including high-value targets like antibodies, will enter the ER via a *sec* translocon [2]. The translocon subunits Sec62p, Sec63p, Sec66p and Sec72p are required for the translocation of certain proteins, particularly those with shorter or less hydrophobic signal peptides [21, 25, 36]. Molecular chaperones are also implicated in protein translocation, through binding of proteins in the cytoplasm (Ssa1p) [20] or the ER lumen (Kar2p) [84]. However, many gene products are able to associate with more than one class of translocon [25, 36]. In addition, while recent structural work suggests that the heptameric Sec61 complex cannot directly bind a ribosome [91, 92], there is a preponderance of evidence demonstrating that the proteins dependent on this complex are translated at the ER membrane [24, 35, 36, 93, 94]. Further, even if a protein does not strictly require particular machinery, like SRP, it may nonetheless sequester it in vivo, reducing availability for proteins that do require these factors [35, 93]. Because of these complexities, it is unsurprising that it has remained difficult to precisely tune a translocon for a specific engineered protein. Rather, optimization will likely require understanding the needs of the target, what the target will sequester, and how this will relate to the balance of resources in the host.

Our calculations for nascent chains produced, ribosomes used, and predicted translocation pathways suggest that each gene presents a unique combination of challenges to the cellular biosynthetic capacity. For instance, long, cotranslationally translocated proteins will impart little demand on cytoplasmic chaperones, but will sequester ribosomes, translocons, and lumenal chaperones for extended periods of time (Fig. 8a). However, because of sustained translation on the surface of the ER, fewer instances of SRP targeting are required. A shorter cotranslational protein will require fewer ribosomes, translocons, and lumenal chaperones to produce the same number of polypeptide chains. However, if the gene is short enough to fail to sustain translation at the membrane (Figs. 5, 8b), then it may require multiple rounds of SRP targetting to get there. If sufficient nascent chains are exposed to the cytosol, the gene may also require cytosolic chaperones. If translation terminates prior to membrane attachment, then posttranslational translocons may be needed as well. Long, posttranslationally translocated proteins will also sequester ribosomes, but will require both lumenal and cytosolic chaperones (Fig. 8c). There are few genes in *K. phaffii* in this category (Fig. 5). Finally, short, posttranslationally translocated proteins will sequester few ribosomes, no cotranslational translocons, and some cytosolic and lumenal chaperones. Our experimental approach cannot measure transit time through posttranslational translocons; we speculate that it will be correlated to polypeptide length.

Some resources used in biogenesis of ER proteins are dependent on chain number, rather than elongation time. For instance, GPI-anchored proteins each receive a single lipid anchor [95], retrograde transport is mediated by the K/HDEL recognition [96], and protein sorting in the secretory pathway involves interactions between cargo and receptors, such as Sec24p [97]. In optimizing these systems, cTPM may be the appropriate metric to consider, and strain engineering efforts could focus on deleting or downregulating highly expressed host proteins. In yeasts, GPI-anchored cell wall proteins present the greatest burden by cTPM. Other aspects are dependent on total polypeptide length, such as the potential ratcheting mechanism provided by Kar2p during translocation [84]. Although not considered here, cTPM scaled by protein length may be the appropriate metric used in engineering. A third aspect is the availability of resources such as ribosomes or translocons, which are sequestered while in operation. cRPM is an appropriate metric to understand ribosome sequestration. For cotranslational translocation, we propose that cRPM could be used as a proxy, as one ribosome binds one translocon during import. In *S. cerevisiae* and *K. phaffii*, expression of *PMA1* appears to be a major ribosome sink, and therefore also a translocon sink. In *K. phaffii*, *PST1* is a second major sink for ribosomes and translocons.

Although fungi are genetically and physiologically diverse, most mechanistic knowledge about secretion is derived from studies in *S. cerevisiae* [2]. Based on a recent molecular dating using 332 genomes [98], *K. phaffii* and *S. cerevisiae* diverged roughly 230 million years ago, whereas the *S. cerevisiae* whole-genome duplication occurred roughly 90 million years ago. Thus, sequence variation is found in nearly all of the proteins conserved in the two species, and due to the paralogy in *S. cerevisiae*, additional differences exist in the regulation of gene expression. Our comparison of *K. phaffii* and *S. cerevisiae* suggests that the path a conserved protein takes to the ER is not necessarily the same between species, even for essential genes critical to health of the secretory pathway, like *PDI1*. However, we find that even though the number and diversity of genes differ between the species, categorically there is conservation in the biosynthetic demand. For instance, our data suggest that *K. phaffii* can provide more nuanced engineering of the cell wall, as it is composed by a greater number of genes. Optimizing fungal species separately may increase protein secretion yields in ways not predicted through analysis of model organisms alone. These results call for a more thorough understanding of industrially used fungal secretion systems for rationally engineering cellular factories during bioproduction.

### Conclusions

Protein biogenesis is a complex phenomena that not only requires raw materials (energy and amino acids), but also access to specialized cellular machinery. Our analysis in *K. phaffii* reveals several principles about these pathways that will be useful in strain engineering. First, we find that a small number of host genes are responsible for most of the protein entering the secretory pathway. Second, GPI-anchored protein components of the cell wall represent the greatest number of nascent chains within the secretory pathway. Third, cotranslational translocation pathways must accommodate a wider set of proteins than posttranslational pathways. Fourth, orthologs may enter the endoplasmic reticulum through different translocation pathways. Fifth, despite differences in the number of genes associated with biological function, the amount of nascent chains entering the ER are similar between *K. phaffii* and *S. cerevisiae*. Finally, we provide an updated genome annotation based on both Ribo-seq and long-read RNA-seq.

## Supplementary Information

**Additional file 1: Figure S1.** Ribo-Seq models active translation. a. Distribution of reads for different length RNA fragments. b. P-site offset for 30 nt fragment reveals active translation.

**Additional file 2: Figure S2.** Ribo-seq and long-read RNA-seq improve transcriptome annotation. Images are screen captures from Integrated Genome Viewer (MIT). a. Ribo-seq reads are stranded. In the top register, ribosome-protected footprint reads mapped to transcripts translated left to right are in red, and reads mapped transcripts translighted right to left are in blue. The middle register shows a prior annotation of transcripts and ORFs. The arrows indicate genes where the annotated translational start site disagrees with Ribo-seq. In both cases, an alternate start codon is used. The bottom register shows the annotation developed here using RNA-seq and long-read RNA-seq data. b. In an example transcript, Ribo-seq (top register) and long-read RNA-seq (bottomregister) reveal both the open reading frame and the untranslated regions (UTRs). c. Flow-chart of the annotation pipeline.

**Additional file 3** Annotation of *K. phaffii* GS115 transcriptome. GFF3 annotation file containing transcript structures derived from Ribo-seq and long-read RNA-seq analysis.

**Additional file 4.** Comparison with prior *K. phaffii* annotations. Comma seperated value (CSV) file that links open reading frames defined in the current study with prior annotations of the *K. phaffii* transcriptome. Includes predicted protein sequence from each annotation. Additional details are provided in the file header.

**Additional file 5.** Combined results of Ribo-seq analysis of *K. phaffii*. Read counts, corrected transcripts per million (cTPM) and corrected ribosomes per million (cRPM) scores for *K. phaffii* GS115 open reading frame (ORFs). The file also includes experimental membrane enrichment scores, predictions of ORF features (localization, signal peptides, GPI-anchors and transmembrane domains), ORF sequences, homology to *S. cerevisiae*, and associations with prior annotations of the *K. phaffii* transcriptome. Additional details are provided in the file header.

**Additional file 6: Figure S3.** Comparison of membrane enrichment between data sets. a. Comparing membrane enrichment in two Ribo-Seq data sets in *K. phaffii*. b. Comparing membrane enrichment in Ribo-Seq data sets in *K. phaffii* and *S. cerevisiae*.

**Additional file 7: Table S1.** Biosynthetic demands for proteins with unknown functions by predicted subcellular localization.

**Additinal file 8.** Combined results of Ribo-seq analysis of *S. cerevisiae*. Read counts, corrected transcripts per million (cTPM) and corrected ribosomes per million (cRPM) scores for *S. cerevisiae* open reading frame (ORFs). The file also includes experimental membrane enrichment scores, predictions of ORF features (localization, signal peptides, GPI-anchors and transmembrane domains), and ORF sequences. Additional details are provided in the file header.

**Additional file 9: Figure S4.** Comparison of metabolic burden for *K. phaffii* and *S. cerevisiae*. a. Total nascent chains for *K. phaffii.* b. Total nascent chains for *S. cerevisiae.*

## Data Availability

The datasets generated and analysed during the current study are available as NCBI Bioproject PRJNA669501.
