# Supplementary figures and images for: Translational landscape and protein biogenesis demands of the early secretory pathway in Komagataella phaffii
Source: Microb Cell Fact. 2021 Jan 20;20:19. doi: 10.1186/s12934-020-01489-9 (PMC7816318; doi:10.1186/s12934-020-01489-9)

Supplemental Figure 1

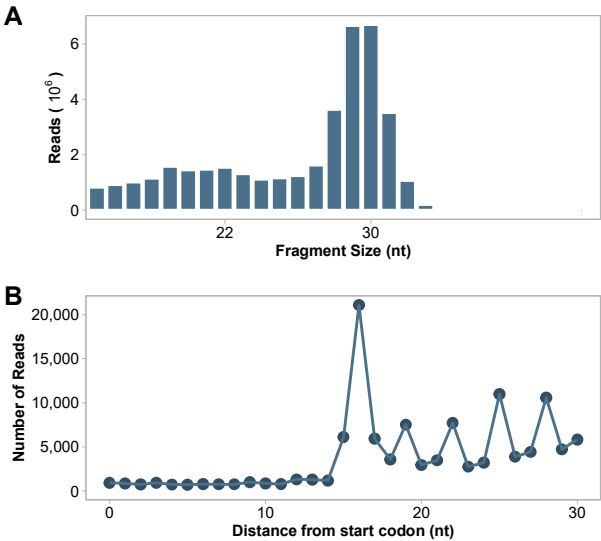

Supplement: Supplementary file 1 — Additional file 1: Figure S1. Ribo-Seq models active translation. a. Distribution of reads for different length RNA fragments. b. P-site offset for 30 nt fragment reveals active translation. [file 12934_2020_1489_MOESM1_ESM.pdf]

Supplemental Figure 2

A

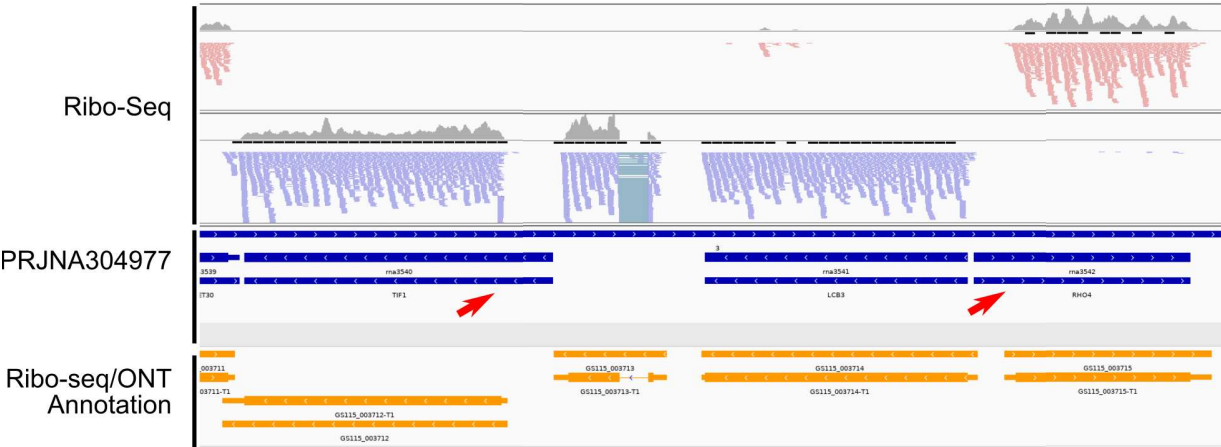

B

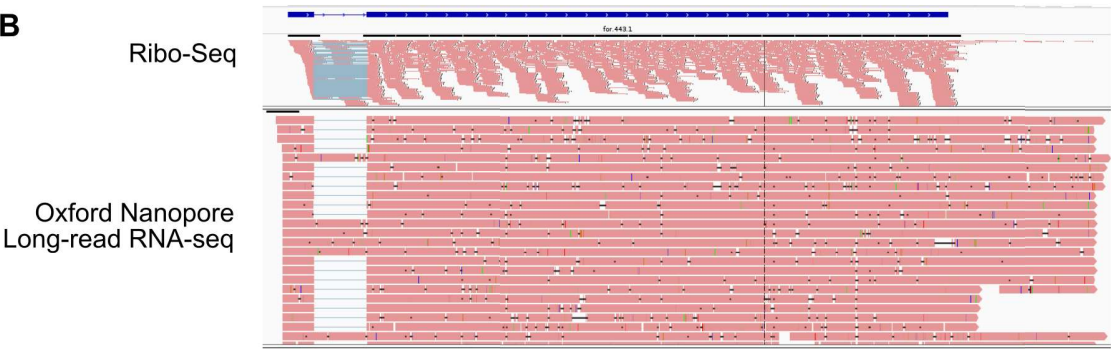

C

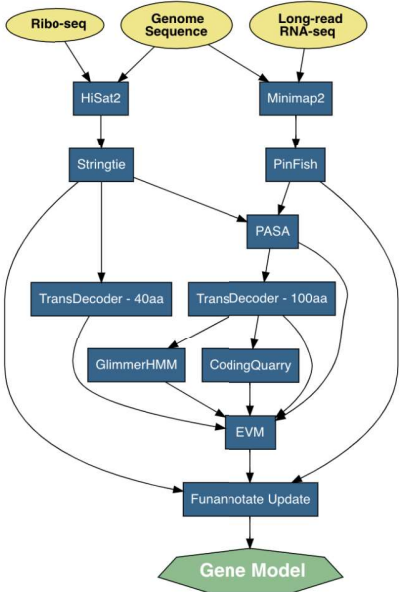

Supplement: Supplementary file 2 — Additional file 2: Figure S2. Ribo-seq and long-read RNA-seq improve transcriptome annotation. Images are screen captures from Integrated Genome Viewer (MIT). a. Ribo-seq reads are stranded. In the top register, ribosome-protected footprint reads mapped to transcripts translated left to right are in red, and reads mapped transcripts translighted right to left are in blue. The middle register shows a prior annotation of transcripts and ORFs. The arrows indicate genes where the annotated translational start site disagrees with Ribo-seq. In both cases, an alternate start codon is used. The bottom register shows the annotation developed here using RNA-seq and long-read RNA-seq data. b. In an example transcript, Ribo-seq (top register) and long-read RNA-seq (bottomregister) reveal both the open reading frame and the untranslated regions (UTRs). c. Flow-chart of the annotation pipeline. [file 12934_2020_1489_MOESM2_ESM.pdf]

Supplemental Figure 3

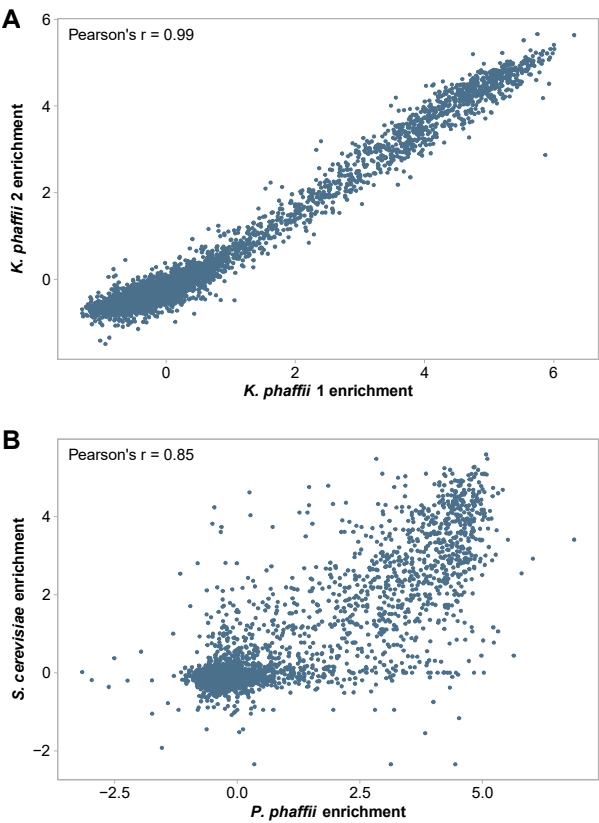

Supplement: Supplementary file 6 — Additional file 6: Figure S3. Comparison of membrane enrichment between data sets. a. Comparing membrane enrichment in two Ribo-Seq data sets in K. phaffii. b. Comparing membrane enrichment in Ribo-Seq data sets in K. phaffii and S. cerevisiae. [file 12934_2020_1489_MOESM6_ESM.pdf]

Supplemental Figure 4

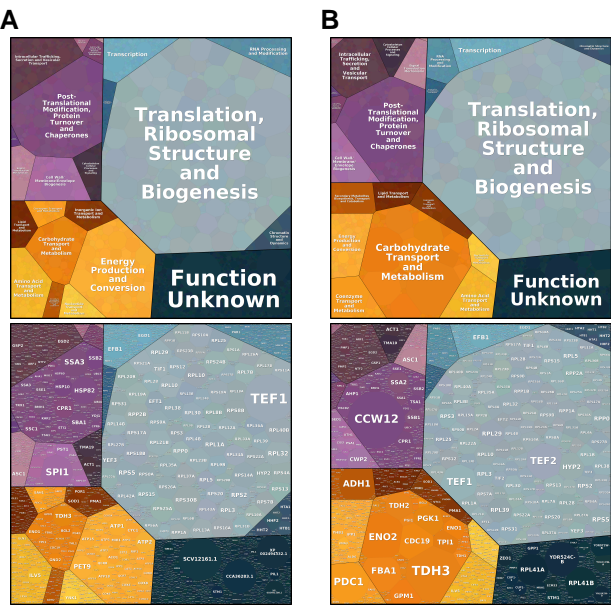

Supplement: Supplementary file 9 — Additional file 9: Figure S4. Comparison of metabolic burden for K. phaffii and S. cerevisiae. a. Total nascent chains for K. phaffii. b. Total nascent chains for S. cerevisiae. [file 12934_2020_1489_MOESM9_ESM.pdf]
