# Supplementary material for: Translational landscape and protein biogenesis demands of the early secretory pathway in Komagataella phaffii
Source: Microb Cell Fact. 2021 Jan 20;20:19. doi: 10.1186/s12934-020-01489-9 (PMC7816318; doi:10.1186/s12934-020-01489-9)
Supplement: Supplementary file 7 — Additional file 7: Table S1. Biosynthetic demands for proteins with unknown functions by predicted subcellular localization. [file 12934_2020_1489_MOESM7_ESM.pdf]

Supplemental Tables

Table S1 Biosynthetic demands for proteins with unknown functions by predicted subcellular localization

	Genes (n)	Mean length (aa)	Nascent chains[a] (%)	Ribosomes[b] (%)
**Cotranslationally Translocated[c]**				
Endoplasmic reticulum	113	446	7.0%	19.0%
Cell membrane	56	494	6.0%	15.0%
Lysosome/Vacuole	30	482	2.0%	7.0%
**Posttranslationally Translocated[d]**				
Extracellular	13	246	79.0%	44.0%
Cell membrane	9	267	2.0%	3.0%
Endoplasmic reticulum	7	453	0.0%	1.0%

[a] Calculated as percent of total cTPM for all proteins predicted to be ER destined.
[b] Calculated as percent of total cRPM for all proteins predicted to be ER destined.
[c] Proteins with greater than 2-fold membrane enrichment and not predicted to be mitochondrial.
[d] Proteins with less than 2-fold membrane enrichment and not predicted to be mitochondrial and contained a predicted signal sequence.
